# Jingfang Granules for prevention of alcohol-induced hangover: a randomized, double-blind, crossover trial in healthy volunteers

**DOI:** 10.3389/fphar.2026.1717099

**Published:** 2026-05-13

**Authors:** Feng Wu, Yuyang Dai, Siyang Ni, Jing Zhang, Xiaojun Hu, Ying Han, Ying Jiao, Huan Lu, Laichun Lu

**Affiliations:** National Institute for Drug Clinical Trial, Beijing Tongren Hospital, Capital Medical University, Beijing, China

**Keywords:** alcohol, hangover, Jingfang Granules, randomized controlled trial, traditional Chinese medicine

## Abstract

**Introduction:**

Jingfang Granules (JFG), a classical traditional Chinese medicine formula comprising 11 botanical drugs, have traditionally been prescribed to dispel cold and eliminate dampness. This study aimed to clinically evaluate the efficacy and safety of JFG in accelerating alcohol clearance and mitigating hangover symptoms.

**Methods:**

A randomized, double-blind, two-period crossover trial enrolled 48 healthy adults. Participants received JFG (6 sachets × 15 g) or placebo 30 min before consuming 100 mL of 56% ABV baijiu within 10 min. Primary endpoints were plasma alcohol concentrations measured up to 24 h post-consumption. Secondary endpoints included plasma alcohol AUC_0–24 h_, plasma alcohol dehydrogenase (ADH) and acetaldehyde dehydrogenase (ALDH) activities, hangover severity assessed using the Acute Hangover Scale (AHS), urine output, and safety.

**Results:**

JFG significantly reduced plasma alcohol concentrations from 30 min to 8 h after consumption (P < 0.005) and decreased plasma alcohol AUC_0–24 h_ (P < 0.001) compared with placebo. AHS scores were numerically lower following JFG treatment; however, the differences were not statistically significant (P > 0.05). JFG administration was associated with greater urine volume (P < 0.001) and fewer urination episodes (P = 0.032). No severe or serious adverse events were reported.

**Discussion:**

JFG significantly reduced plasma alcohol levels for up to 8 h after consumption and showed a trend toward lower hangover symptom scores; however, the mechanism remains unknown.

**Clinical Trial Registration:**

https://www.chictr.org.cn/, identifier ChiCTR2400084155.

## 1 Introduction

Alcohol is one of the most widely consumed psychoactive substances worldwide and is commonly used for social and recreational purposes. According to the Global Status Report on Alcohol and Health (World Health Organization, 2024), global *per capita* consumption in 2019 was estimated at 5.5 L of pure alcohol among individuals aged 15 years and older. Harmful use of alcohol is a major public health concern, contributing to approximately 2.6 million deaths each year. Alcohol plays a causal or contributory role in more than 230 diseases and health conditions. Among drinkers, the average *per capita* intake is approximately 27 g of pure alcohol per day, a level associated with substantially increased risks of multiple diseases, mortality, and disability (Ding et al., 2021).

In addition, alcohol intoxication remains a global health burden, with limited effective detoxification agents available. Several commonly consumed products, including coffee, tea, fluids, vitamin B_6_, and painkillers, are often recommended to alleviate hangover symptoms. However, even those ingredients that demonstrate some beneficial effects against acute alcohol toxicity exhibit limited efficacy in accelerating alcohol metabolism and relieving hangover symptoms (Jayawardena et al., 2017). For example, survey data indicate that water consumption during or after alcohol intake is ineffective in alleviating alcohol hangovers (Mackus et al., 2024).

In China, Jingfang Granules (JFG) is a modern Chinese botanical drug derived from Jingfang Baidu Powder, a formulation recorded in a Ming Dynasty (1,368–1,644) text and originally prescribed to dispel cold and eliminate dampness. The documented therapeutic effects of Jingfang Baidu Powder include releasing the exterior and warming the interior, dispelling external wind, and eliminating dampness. JFG is currently used in the treatment of acute viral upper respiratory tract infections and early stages of plague (Yue et al., 2025). Within the framework of traditional Chinese medicine, excessive alcohol intake is believed to generate damp-heat, which is associated with systemic inflammation and neuropsychiatric symptoms such as dizziness, insomnia, and agitation. Accordingly, the use of JFG to alleviate or prevent alcohol-induced hangover may represent a rational therapeutic approach.

Preclinical evidence suggests the potential mechanisms of JFG in relieving alcohol intoxication and protecting the liver in mice. Using network pharmacology and molecular docking, one study showed that JFG may enhance alcohol metabolism, possibly through multiple components and targets (Gao et al., 2021). Another study suggested that JFG may exert an antidotal effect in mice by increasing the activity or expression of alcohol-metabolizing enzymes such as alcohol dehydrogenase (ADH), catalase, and acetaldehyde dehydrogenase (ALDH) (Gao et al., 2023).

Despite these preclinical findings, clinical evidence for JFG’s sobering effects in humans is lacking. Therefore, this exploratory study aimed to investigate the efficacy of JFG in reducing blood alcohol concentration and promoting sobriety using a rigorous crossover design, thereby providing a translational link between ethnopharmacological knowledge and modern evidence-based practice.

## 2 Materials and methods

### 2.1 Study design

**FIGURE 1:**
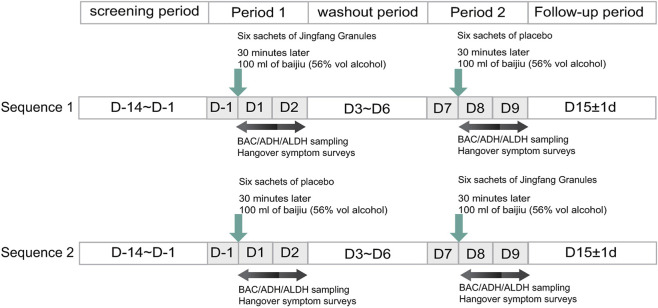
Study design. BAC, Blood Alcohol Concentration; ADH, Alcohol Dehydrogenase; ALDH, Aldehyde Dehydrogenase.

This was a single-center, randomized, double-blind, two-period crossover trial conducted at Beijing Tongren Hospital, Capital Medical University. Participants were randomly assigned in a 1:1 ratio to receive JFG followed by placebo, or placebo followed by JFG. Each intervention was separated by a 7-day washout interval (Figure 1). To ensure blinding integrity, the JFG and placebo preparations were identical in appearance and packaging. Randomization sequences were computer-generated using SAS version 9.4 (SAS Institute Inc., Cary, NC, United States).

This study was approved by the Ethics Committee of Beijing Tongren Hospital, Capital Medical University (Approval No. TREC2023-124) and conducted in accordance with the ethical principles of the Declaration of Helsinki and International Council for Harmonization Good Clinical Practice guidelines. Written informed consent was obtained from all participants before initiation of any study procedures.

### 2.2 Participants

Healthy adults aged 18–60 years with a body mass index (BMI) of 19–26 kg/m^2^ were enrolled. Before enrollment, participants underwent a medical history interview followed by a routine physical examination, including assessment of vital signs, 12-lead electrocardiograms (ECGs), and laboratory tests (hematology, blood biochemistry, urinalysis, and screening for HIV, hepatitis B surface antigen, and anti-hepatitis C virus antibodies) to confirm eligibility.

Exclusion criteria included allergy to any components of JFG or its 11 botanical drugs, alcohol allergy, smoking more than five cigarettes per day, hepatic or renal impairment, recent medication use, and abnormal urine screening results. Participants were also excluded if they met either of the following conditions: 1) daily alcohol consumption of ≥200 mL of 56% alcohol by volume (ABV) baijiu without experiencing intoxication symptoms, or 2) manifestation of intoxication symptoms after consuming ≤25 mL of 56% ABV baijiu. Intoxication symptoms were defined as varying degrees of excitement or agitation, loss of behavioral restraint, abnormal conduct, talkativeness with slurred speech, motor and gait incoordination, aggression, or drowsiness.

### 2.3 Intervention

JFG is composed of 11 botanical drugs, including *Schizonepeta tenuifolia* Briq. [Lamiaceae; *Schizonepetae herba*], *Saposhnikovia divaricata* (Turcz.) Schischk. [Apiaceae; *Saposhnikoviae radix*], *Bupleurum chinense* DC. [Apiaceae; *Bupleuri radix*], *Ligusticum chuanxiong* Hort. [Apiaceae; *Chuanxiong Rhizoma*], *Notopterygium incisum* Ting ex H. T. Chang [Apiaceae; *Notopterygii Radix et Rhizoma*], *Angelica pubescens* Maxim. f. biserrata Shan et Yuan [Apiaceae; *Angelicae Pubescentis Radix*], *Peucedanum praeruptorum* Dunn [Apiaceae; *Peucedani Radix*], *Poria cocos* (Schw.) Wolf [Polyporaceae; *P. cocos*], *Citrus aurantium* L. [Rutaceae; *Aurantii Fructus Immaturus*], *Platycodon grandiflorus* (Jacq.) A. DC. [Campanulaceae; *Platycodonis Radix*], and *Glycyrrhiza uralensis* Fisch. [Fabaceae; *Glycyrrhizae Radix et Rhizoma*], at a dry weight ratio of 3:3:3:3:3:3:3:3:3:3:1, respectively. No restricted substances or botanical drugs derived from endangered species were used. All materials were authenticated by Senior Engineer Jianwei Fan (Chinese National Key Laboratory of Classical Prescription and Modern Chinese Medicine Integration), and voucher specimens were deposited in the institutional repository. JFG was processed through extraction, percolation, filtration, and concentration following the procedures listed in the Pharmacopoeia of the People’s Republic of China, 2025 (Chinese Pharmacopoeia Commission, 2025). The chemical composition of JFG was characterized by GC–MS and UPLC–Q-Exactive MS in both positive and negative ion modes. A total of 24 compounds (19 terpenoids, 2 aldehydes, 2 esters, and 1 aromatic ether) were identified by GC–MS, and 85 compounds (25 coumarins, 6 lignans, 15 flavonoid glycosides, 15 flavonoids, 9 organic acids, 5 glycosides, 4 triterpenoids, and 6 other compounds) were identified by UPLC–Q-Exactive MS, based on reference standards, literature, and database retrieval (Liang et al., 2022).

JFG and placebo were both provided by Lunan Hope Pharmaceutical Co., Ltd. (Linyi, China) in accordance with Good Manufacturing Practice and relevant regulatory requirements. JFG consisted of botanical drug extracts, mannitol, water, and double caramel color in a ratio of 1:20:5.5:4.5, whereas the placebo replaced the botanical drug extracts with mannitol to match taste and appearance. Batch analysis records were retained. The investigational product (batch no. 0012301074) was supplied as 15 g per sachet.

During the first study period, participants fasted for 10 h before receiving either JFG or placebo, followed by consumption of 100 mL of baijiu (56% ABV)) within 10 min. In the second period, the same protocol was applied, with treatments crossed over between the two groups. Each dose of JFG or placebo (6 sachets × 15 g) was dissolved in 100 mL of warm boiled water (50 °C–60 °C) and administered orally 30 min before alcohol intake. All interventions were performed under medical supervision.

To minimize potential confounding effects, participants were instructed to abstain from alcohol for at least 1 week before each intervention. In addition, before each intervention, medical history was reviewed, and all participants underwent a complete physical examination and an alcohol breath test.

### 2.4 Plasma alcohol, ADH, and ALDH levels

Venous blood samples were collected at 15, 30, and 45 min and at 1, 2, 4, 8, and 24 h after alcohol consumption into EDTA-K_2_-treated tubes. Samples were centrifuged at 1,700 × *g* for 10 min at 2 °C–8 °C and stored at −80 °C until analysis. Plasma was used to determine alcohol concentration as well as ADH and ALDH activities.

As described in previous studies (Musile et al., 2023; Wachełko et al., 2021), plasma alcohol concentration was analyzed using headspace gas chromatography coupled with a flame ionization detector. ADH and ALDH activities were determined using an enzymatic assay kit (Beijing Bio-leader Biology Co., China), and optical density was measured at 340 nm using a microplate reader (Tecan Group Ltd., Switzerland).

### 2.5 Survey of hangover symptoms

Hangover severity was assessed using the Acute Hangover Scale (AHS) (Rohsenow et al., 2007; Verster et al., 2020). The scale includes nine items: “hangover,” “thirsty,” “tired,” “headache,” “dizziness/faintness,” “loss of appetite,” “stomachache,” “nausea,” and “heart racing.” Each item is rated from 0 to 7, with anchors defined as “none” [0], “mild” [1], “moderate” [4], and “incapacitating” [7]. Overall hangover severity is calculated as the sum of the nine item scores. Assessments were performed at 2 and 24 h after alcohol consumption by the same physician to ensure consistency.

In addition, total water intake, number of urinations, and total urine volume were recorded from drug administration until 8 h after alcohol consumption. Urine was collected in graduated containers, water intake was measured using calibrated bottles, and fluid intake was standardized across participants during the monitoring period.

### 2.6 Safety assessment

Pharmacovigilance monitoring over 10 years identified 551 individual case reports (25 serious cases) of suspected adverse drug reactions (ADRs) related to JFG, involving 845 adverse events, mainly affecting the gastrointestinal system (54.1%), skin and subcutaneous tissues (24.3%), and nervous system (5.4%). The estimated reporting rate was approximately 0.16 cases per 100,000 treatment courses. Therefore, these parameters were carefully monitored. Safety assessments were conducted according to predefined schedules and included monitoring of adverse events (AEs), vital signs, 12-lead ECGs, laboratory parameters, and physical examinations. Baseline evaluations were performed prior to initial drug administration, and all participants underwent continuous monitoring throughout the study. Investigators assessed the occurrence of AEs, their severity according to CTCAE v5.0, and their relationship to the study drug using WHO–UMC criteria.

### 2.7 Statistical analysis

Participants who were successfully randomized and received at least one dose of JFG or placebo were included in the full analysis set (FAS). Those who demonstrated adequate adherence to the study protocol and had no major protocol deviations constituted the per-protocol set (PPS). The safety set (SS) consisted of all randomized participants who received at least one treatment and had available post-treatment safety data. Primary efficacy analyses were performed in both the FAS and PPS populations, whereas safety analyses were conducted in the SS population.

Categorical variables are summarized as percentages or proportions. Continuous variables are summarized as mean ± standard deviation (SD), or median (range: minimum–maximum), as appropriate. Statistical analyses were conducted using SAS version 9.4. Paired two-sided *t*-tests were used to compare outcomes between the JFG and placebo groups, with P < 0.05 considered statistically significant. To control the inflation of type I error caused by multiple comparisons, the Bonferroni correction method was applied. The corrected significance level was calculated as 0.05 divided by the total number of tests. A P value <0.00625 was considered statistically significant for comparisons of alcohol concentrations at multiple time points after administration. Descriptive statistic was the primary method used for safety evaluation.

The area under the concentration–time curve from time zero to the last measurable concentration (AUC_0–t_) for plasma alcohol was determined using a non-compartmental model with Phoenix WinNonlin 8.3 (Pharsight Corporation, Sunnyvale, CA, United States).

## 3 Results

### 3.1 Participants characteristics

A total of 48 participants were randomized, received at least one dose of the investigational product, and were included in the FAS and SS. Among them, eight participants discontinued prematurely owing to incomplete alcohol consumption or alcohol-induced vomiting. The remaining 40 participants (83.3%) completed both study periods per protocol and were included in the PPS.

Baseline demographic characteristics of the FAS population are summarized in Table 1. All participants were of Han Chinese ethnicity, with a mean age of 31.2 years and a mean BMI of 22.7 kg/m^2^, within the normal range. Of the 48 participants, 38 (79.2%) were men. Demographic characteristics were comparable between the two treatment sequences.

**TABLE 1:** Demographic characteristics.

Outcomes	Sequence JFG-placebo (N = 24)	Sequence Placebo-JFG (N = 24)	Total (N = 48)
Sex, male	18 (75.0%)	20 (83.3%)	38 (79.2%)
Sex, female	6 (25.0%)	4 (16.7%)	10 (20.8%)
Age (years)	30.3 (10.1)	32.1 (12.7)	31.2 (11.4)
Height (cm)	170.0 (5.9)	168.7 (7.7)	169.4 (6.8)
Weight (kg)	65.5 (6.9)	65.1 (7.6)	65.3 (7.2)
Body mass index (kg/m^2^)	22.7 (2.0)	22.8 (1.5)	22.7 (1.7)

Data is presented as mean (standard deviation), except for sex, which is presented as number (%).

At baseline, all participants had normal findings on physical examination, vital signs, and ECGs. Some participants presented with abnormal laboratory results; however, these were considered clinically insignificant by the investigator.

### 3.2 Plasma alcohol, ADH, and ALDH levels

**FIGURE 2:**
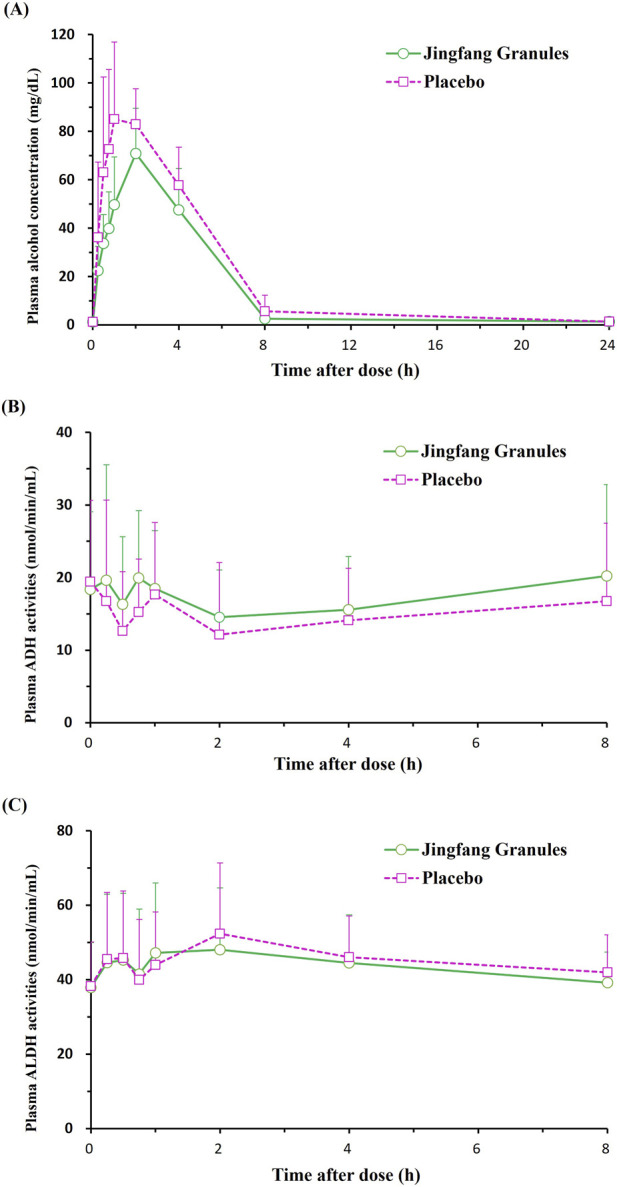
Mean plasma concentration-time profiles of **(A)** alcohol, **(B)** ADH, and **(C)** ALDH. ADH: alcohol dehydrogenase; ALDH: acetaldehyde dehydrogenase. Linear scales. Error bars represent standard deviations.

Plasma alcohol concentrations were significantly lower following JFG administration than placebo at multiple post-consumption time points: 30 min [33.7 (12.0) mg/dL vs. 63.0 (39.5) mg/dL, P < 0.001], 45 min [39.9 (15.1) mg/dL vs. 72.7 (32.8) mg/dL, P < 0.001], 1 h [49.7 (19.8) mg/dL vs. 85.1 (31.8) mg/dL, P < 0.001], 2 h [70.9 (18.7) mg/dL vs. 82.9 (14.7) mg/dL, P < 0.001], 4 h [47.5 (17.2) mg/dL vs. 57.7 (15.8) mg/dL, P < 0.001], and 8 h [2.5 (1.3) mg/dL vs. 5.6 (6.7) mg/dL, P = 0.003] after alcohol consumption (Figure 2A).

No significant differences were observed between the two treatments at baseline [1.2 (0.4) mg/dL vs. 1.2 (0.3) mg/dL, P = 0.776] or at 24 h [1.4 (0.3) mg/dL vs. 1.4 (0.4) mg/dL, P = 0.369] after alcohol consumption (Figure 2A).

Overall alcohol exposure, assessed by the AUC, was significantly reduced with JFG [339.6 (93.0) hmg/dL] compared with placebo [462.1 (121.6) hmg/dL, P < 0.001] (Figure 2A).

Plasma ADH activity was significantly higher following JFG administration than placebo at 45 min post-alcohol intake [20.0 (9.3) nmol/min/mL vs. 15.3 (7.3) nmol/min/mL, P = 0.005] (Figure 2B). Although ADH activity tended to be higher in the JFG group at other time points (15 and 30 min, and 1, 2, 4, and 8 h), these differences did not reach statistical significance (P > 0.05) (Figure 2B). Similarly, plasma ALDH activity did not differ significantly between the two groups at baseline (0 min) or at any subsequent time point (P > 0.05) (Figure 2C). Detailed ADH and ALDH activity data are provided in Table 2.

**TABLE 2:** Plasma ADH and ALDH activities over 8 h following alcohol intake with Jingfang Granules and placebo treatment.

Time	ADH activities (nmol/min/mL)			ALDH activities (nmol/min/mL)		
	Jingfang Granules	Placebo	P	Jingfang Granules	Placebo	P
0	18.4 (10.7)	19.4 (11.2)	0.932	37.9 (12.1)	38.3 (11.8)	0.850
15 min	19.6 (15.9)	16.8 (13.9)	0.587	44.6 (18.3)	45.5 (18.0)	0.935
30 min	16.3 (9.3)	12.6 (8.2)	0.132	45.2 (17.9)	45.8 (18.1)	0.822
45 min	20.0 (9.3)	15.3 (7.3)	0.005	41.5 (17.4)	40.0 (16.2)	0.768
1 h	18.5 (8.0)	17.6 (10.0)	0.844	47.2 (18.8)	44.0 (14.2)	0.282
2 h	14.5 (6.5)	12.1 (10.0)	0.107	48.1 (16.6)	52.4 (19.0)	0.342
4 h	15.6 (7.3)	14.1 (7.2)	0.557	44.5 (12.9)	46.0 (11.1)	0.628
8 h	20.2 (12.6)	16.8 (10.7)	0.330	39.2 (8.2)	42.0 (10.0)	0.193

Data are presented as mean ± standard deviation. Analyses are based on the FAS, populations (N = 48).

ADH: alcohol dehydrogenase; ALDH: acetaldehyde dehydrogenase.

The analyses presented above are based on the FAS (N = 48). Analyses of the PPS (N = 40) demonstrated consistent findings across all efficacy endpoints, including plasma alcohol concentration and ADH/ALDH activity profiles.

### 3.3 Urine output

No significant between-group difference was observed in total water intake during the 8-h post-intervention period [JFG: 661.7 (117.2) mL vs. placebo: 686.7 (56.1) mL; P = 0.083, FAS population]. This finding suggests that hydration status was comparable between the treatments. JFG treatment resulted in fewer urination episodes than placebo [2.6 (1.0) vs. 3.3 (2.2); P = 0.032). In contrast, total urine volume over the 8-h post-intervention period was higher following JFG treatment than placebo [740.5 (260.4) mL vs. 252.4 (147.0) mL; P < 0.001].

Analyses based on the PPS population demonstrated consistent findings for water intake, urination episodes, and total urine output.

### 3.4 Survey of hangover symptoms

JFG demonstrated numerically lower AHS scores than placebo at both 2 h [6.1 (5.7) vs. 7.1 (6.1)] and 24 h [0.2 (0.7) vs. 0.3 (0.83)] after alcohol consumption; however, these differences were not statistically significant (P = 0.259 and P = 0.884, respectively, FAS population). Analyses of the PPS population confirmed consistent findings.

### 3.5 Adverse events

In the SS population, 11 treatment-emergent adverse events (TEAEs) were reported by 10 participants following JFG treatment, whereas 40 TEAEs occurred in 24 participants who received placebo. The incidence of AEs differed significantly between the two groups (20.8% vs. 50.0%; P = 0.003). No treatment-related adverse events (TRAEs) were observed following JFG administration, whereas 21 participants in the placebo group (43.8%) experienced 27 TRAEs (P < 0.001). A summary of all TEAEs is presented in Table 3.

**TABLE 3:** Treatment-emergent adverse events in safety analysis.

Preferred term of TEAEs	Jingfang Granules (N = 48)	Placebo (N = 48)
Gastrointestinal disorders		
Diarrhea^*^	0	21 (43.8%)
Abdominal pain^*^	0	4 (8.3%)
Investigations		
Elevated triglycerides	7 (14.6%)	1 (2.1%)
Proteinuria	1 (2.1%)	2 (4.2%)
Hypotension	1 (2.1%)	0
Increased blood creatinine	1 (2.1%)	0
Elevated total cholesterol	0	1 (2.1%)
Elevated LDL cholesterol	0	1 (2.1%)
Elevated fibrinogen	0	1 (2.1%)
Abnormal urinary sediment	0	1 (2.1%)
Abnormal urinalysis	0	1 (2.1%)
Leukocyturia	0	1 (2.1%)
Infections		
Acute upper respiratory infection	0	1 (2.1%)
Acute pharyngitis	0	1 (2.1%)
Skin infection^*^	0	1 (2.1%)
Skin disorders		
Papular urticaria^*^	0	1 (2.1%)
Urticaria	0	1 (2.1%)
Blood disorders		
Anemia	0	1 (2.1%)
General disorders		
Fever	1 (2.1%)	0

MedDRA v27.0 coding; Data presented as n (%). LDL: Low density lipoprotein; TEAEs: Treatment-emergent adverse events.

* Assessed as potentially treatment-related.

No AEs of CTCAE grade ≥3 severity or serious AEs were observed during either treatment period. All AEs resolved completely during the study period, except for three events with unknown outcomes due to loss to follow-up for personal reasons.

## 4 Discussion

As JFG has shown potential effects in enhancing alcohol detoxification in preclinical studies, we conducted a randomized, double-blind, two-period crossover study to further investigate its clinical applicability. To our knowledge, this is the first clinical trial to evaluate the efficacy and safety of JFG in accelerating alcohol elimination and promoting sobriety in healthy volunteers. The crossover design allows each participant to serve as their own control, thereby reducing variability and increasing statistical power. In addition, because baseline endogenous alcohol levels are less than 0.05 mg/mL and the measured concentrations at time zero in both periods were consistently below this threshold, the washout period was considered adequate. Furthermore, because alcohol metabolism follows zero-order kinetics, the 7-day washout period was sufficient to eliminate prior alcohol intake. Thus, a meaningful carryover effect is unlikely to have influenced the study outcomes.

Although refraining from alcohol remains the most reliable way to prevent hangovers, the development of targeted interventions such as JFG represents a feasible approach to mitigating hangover symptoms and associated functional impairments, including deficits in cognition and memory. Accelerating alcohol metabolism is considered a direct strategy for developing hangover remedies.

Alcohol is metabolized to acetaldehyde by ADH, and acetaldehyde is subsequently oxidized to acetate by ALDH. Therefore, plasma ADH and ALDH activities can serve as systemic markers of alcohol metabolism. In the present study, however, the activities of both enzymes did not differ significantly between the JFG and placebo groups at most time points. Acetaldehyde is highly toxic and has been implicated in the pathogenesis of hangover symptoms such as nausea, sweating, tachycardia, and headache (Edenberg and McClintick, 2018). Because ADH activity was not consistently elevated by JFG, acetaldehyde levels were likely comparable between groups. Nevertheless, JFG may exert its effects through alternative acetaldehyde-related mechanisms. During hangover development, acetaldehyde has been shown to modulate immune responses and promote inflammation, including alterations in cytokine levels (González-Reimers et al., 2014; Kim et al., 2003). It also facilitates histamine release from mast cells and impairs metabolic clearance by inhibiting diamine oxidase activity (Tipple et al., 2016; Zimatkin and Anichtchik, 1999), and it plays a role in the induction of oxidative stress (Cederbaum, 2012). Thus, rather than directly altering ADH or ALDH activity, JFG may interact with downstream pathways involved in acetaldehyde-mediated processes to alleviate hangover symptoms.

Furthermore, the potential involvement of JFG in alternative alcohol metabolic pathways should be considered, as plasma alcohol concentrations were significantly lower in the JFG group than in the placebo group at all time points between 30 min and 8 h, despite no consistent changes in ADH activity. CYP2E1 can also metabolize alcohol to acetaldehyde in the liver, although this pathway is typically more relevant in chronic alcohol use (Lu and Cederbaum, 2008). Notably, JFG has been shown to suppress alcohol-induced CYP2E1 expression in a mouse model of acute alcohol-induced hangover (Gao et al., 2023), suggesting that JFG may also modulate CYP2E1 expression in humans. The accelerated clearance of plasma alcohol observed in the JFG group may therefore reflect enhanced elimination of alcohol and its metabolites through mechanisms yet to be elucidated, potentially involving CYP2E1. It is also possible that JFG reduces plasma alcohol levels through effects on alcohol absorption or distribution. Further studies are warranted to clarify the underlying mechanisms of action.

Although water intake volumes were comparable between groups, the JFG group exhibited significantly greater urine output and fewer urination episodes than the placebo group. However, no studies have directly examined whether diuresis contributes to the elimination of alcohol metabolites, although alcohol itself is known to exert diuretic effects (Grupp et al., 1991). Further investigation may help clarify this potential mechanism, particularly in light of the role of JFG in promoting dampness clearance through diuresis and facilitating diaphoresis within the framework of traditional Chinese medicine.

Although JFG showed non-significant reductions in hangover symptoms at 2 and 24 h alcohol consumption, the consistent reduction in plasma alcohol concentrations suggests potential symptomatic benefits. Similar findings have been reported in previous antidotal trials (Köchling et al., 2019; Mackus et al., 2018) evaluating alcohol concentration and hangover severity Individual factors, such as tolerance, genetic variation (Edenberg and Foroud, 2014; Yokoyama et al., 2014), biochemical alterations (Palmer et al., 2019) and habituation to alcohol intake, are likely to contribute to variability in hangover susceptibility. Future studies with larger sample sizes or optimized designs may help clarify the symptomatic efficacy.

In this study, the absence of severe AEs (grade ≥3) and the complete resolution of all documented events support JFG’s favorable safety profile. The absence of TRAEs with JFG, compared with the placebo (43.8% incidence), further supports its safety. The high incidence of gastrointestinal AEs in the placebo group is likely unrelated to the placebo itself and may instead reflect alcohol-induced mucosal irritation, as participants consumed a large amount of alcohol within a short time on an empty stomach. In addition, the potential gastroprotective properties of JFG, consistent with its traditional use, may have contributed to these findings (Sun et al., 2023; Lv et al., 2023; Zhu et al., 2017; Arab et al., 2019). Although these findings are clinically relevant, they require validation in larger cohorts with dedicated assessments. Furthermore, more realistic scenarios, such as alcohol intake with food or lower-proof beverages over longer periods, should be evaluated in future studies to better reflect real-world conditions.

### 4.1 Study limitations

This study has several limitations. The relatively small sample size may have contributed to the lack of statistical significance in some outcomes, despite the use of a crossover design. In addition, the limited sample size may restrict generalizability and reduce the stability of effect estimates. The use of multiple paired t-tests remains a methodological limitation, despite the application of Bonferroni correction for multiple comparisons. Furthermore, the absence of a formal statistical assessment of potential carry-over effects is also a limitation. Therefore, the findings should be interpreted with caution. Larger studies are required to confirm these results. Only a single, rapidly consumed high dose of alcohol and JFG was evaluated, which may further limit generalizability. To better reflect real-world consumption patterns and to distinguish the general effects of alcohol from those of high-proof alcohol, future studies should include multiple dosing regimens. As in many similar studies, the participant population was predominantly male, raising the possibility of sex-related bias. However, lifestyle factors, such as insufficient physical activity, may exert a stronger influence than sex- or age-related differences. These potential physiological influences warrant further investigation. Finally, additional parameters, including cytokines, inflammatory biomarkers, and alcohol metabolites, should be assessed in future studies to provide deeper insight into the mechanisms by which JFG may alleviate hangover symptoms.

## 5 Conclusion

We demonstrated that JFG significantly reduced plasma alcohol levels for up to 8 h after alcohol consumption; however, the underlying mechanism remains unknown. While JFG showed a trend toward lower hangover symptom scores, this observation did not reach statistical significance and therefore requires further confirmation in future studies.

## Data Availability

The original contributions presented in the study are included in the article/supplementary material, further inquiries can be directed to the corresponding authors.

## References

[B1] Arab H. H. Salama S. A. Eid A. H. Kabel A. M. Shahin N. N. (2019). Targeting MAPKs, NF-κB, and PI3K/AKT pathways by methyl palmitate ameliorates ethanol-induced gastric mucosal injury in rats. J. Cell Physiol. 234, 22424–22438. 10.1002/jcp.28807 31115047

[B2] Cederbaum A. I. (2012). Alcohol metabolism. Clin. Liver Dis. 16, 667–685. 10.1016/j.cld.2012.08.002 23101976 PMC3484320

[B3] Ding C. O'Neill D. Bell S. Stamatakis E. Britton A. (2021). Association of alcohol consumption with morbidity and mortality in patients with cardiovascular disease: original data and meta-analysis of 48,423 men and women. BMC Med. 19, 167. 10.1186/s12916-021-02040-2 34311738 PMC8314518

[B4] Edenberg H. J. Foroud T. (2014). Genetics of alcoholism. Handb. Clin. Neurol. 125, 561–571. 10.1016/B978-0-444-62619-6.00032-X 25307596

[B5] Edenberg H. J. McClintick J. N. (2018). Alcohol dehydrogenases, aldehyde dehydrogenases, and alcohol use disorders: a critical review. Alcohol Clin. Exp. Res. 42 (12), 2281–2297. 10.1111/acer.13904 30320893 PMC6286250

[B6] Gao M. Yang R. C. Liu Q. Lei W. Rao Z. L. Zeng N. (2021). Mechanism of Jingfang Granules in relieving alcohol and protecting liver based on bioinformatics technology. China J. Chin. Mat. Med. 46, 5683–5692. Chinese. 10.19540/j.cnki.cjcmm.20210721.401 34951222

[B7] Gao M. Zhang X. Luo D. M. Yang R. C. Rao Z. L. Zeng N. (2023). Anti-drinking effect and mechanism of Jingfang Granules on acute drunkenness model mice. Chin. Tradit. Herb. Drugs 54, 1164–1172. Chinese. 10.7501/j.issn.0253-2670.2023.04.016

[B8] González-Reimers E. Santolaria-Fernández F. Martín-González M. C. Fernández-Rodríguez C. M. Quintero-Platt G. (2014). Alcoholism: a systemic proinflammatory condition. World J. Gastroenterol. 20 (40), 14660–14671. 10.3748/wjg.v20.i40.14660 25356029 PMC4209532

[B9] Grupp L. A. Perlanski E. Stewart R. B. (1991). Regulation of alcohol consumption by the renin-angiotensin system: a review of recent findings and a possible mechanism of action. Neurosci. Biobehav. Rev. 15 (2), 265–275. 10.1016/S0149-7634(05)80006-5 1852316

[B10] Jayawardena R. Thejani T. Ranasinghe P. Fernando D. Verster J. C. (2017). Interventions for treatment and/or prevention of alcohol hangover: systematic review. Hum. Psychopharmacol. 32, e2600. 10.1002/hup.2600 28568743

[B11] Kim D. J. Kim W. Yoon S. J. Choi B. M. Kim J. S. Go H. J. (2003). Effects of alcohol hangover on cytokine production in healthy subjects. Alcohol 31 (3), 167–170. 10.1016/j.alcohol.2003.09.003 14693266

[B12] Köchling J. Geis B. Wirth S. Hensel K. O. (2019). Grape or grain but never the twain? A randomized controlled multiarm matched-triplet crossover trial of beer and wine. Am. J. Clin. Nutr. 109, 345–352. 10.1093/ajcn/nqy309 30753321 PMC6410559

[B13] Liang H. Jiang Y. Yuan X. Yao J. Qiu R. Yang M. (2022). Chemical constituents of Jingfang Granules based on GC-MS and UPLC-Q exactive MS. Chin. Tradit. Herb. Drugs 53 (6), 1697–1708. Chinese. 10.7501/j.issn.0253-2670.2022.06.012

[B14] Lu Y. Cederbaum A. I. (2008). CYP2E1 and oxidative liver injury by alcohol. Free Radic. Biol. Med. 44 (5), 723–738. 10.1016/j.freeradbiomed.2007.11.004 18078827 PMC2268632

[B15] Lv K. Li M. Sun C. Miao Y. Zhang Y. Liu Y. (2023). Jingfang Granule alleviates bleomycin-induced acute lung injury via CD200-CD200R immunoregulatory pathway. J. Ethnopharmacol. 311, 116423. 10.1016/j.jep.2023.116423 37011735

[B16] Mackus M. van Schrojenstein Lantman M. Van de Loo A. J. A. E. Kraneveld A. D. Garssen J. Brookhuis K. A. (2018). Alcohol metabolism in hangover sensitive versus hangover resistant social drinkers. Drug Alcohol Depend. 185, 351–355. 10.1016/j.drugalcdep.2017.11.040 29500954

[B17] Mackus M. Stock A. K. Garssen J. Scholey A. Verster J. C. (2024). Alcohol hangover versus dehydration revisited: the effect of drinking water to prevent or alleviate the alcohol hangover. Alcohol 121, 9–18. 10.1016/j.alcohol.2024.01.005 39069212

[B18] Musile G. Pigaiani N. Pasetto E. Ballotari M. Tagliaro F. Bortolotti F. (2023). Validation of a new salt-assisted HS-GC-FID method for the determination of ethanol in the vitreous humor. J. Anal. Toxicol. 46, e274–e279. 10.1093/jat/bkac087 36346343

[B19] Palmer E. Tyacke R. Sastre M. Lingford-Hughes A. Nutt D. Ward R. J. (2019). Alcohol hangover: underlying biochemical, inflammatory and neurochemical mechanisms. Alcohol Alcohol 54, 196–203. 10.1093/alcalc/agz016 30916313

[B20] Rohsenow D. J. Howland J. Minsky S. J. Greece J. Almeida A. Roehrs T. A. (2007). The acute hangover scale: a new measure of immediate hangover symptoms. Addict. Behav. 32, 1314–1320. 10.1016/j.addbeh.2006.10.001 17097819 PMC2853365

[B21] Sun C. Liang H. Zhao Y. Li S. Li X. Yuan X. (2023). Jingfang Granules improve glucose metabolism disturbance and inflammation in mice with urticaria by up-regulating LKB1/AMPK/SIRT1 axis. J. Ethnopharmacol. 302, 115913. 10.1016/j.jep.2022.115913 36347302

[B22] Tipple C. T. Benson S. Scholey A. (2016). A review of the physiological factors associated with alcohol hangover. Curr. Drug. Abuse Rev. 9 (2), 93–98. 10.2174/1874473710666170207152933 28176621

[B23] Verster J. C. van de Loo A. J. A. E. Benson S. Scholey A. Stock A. K. (2020). The assessment of overall hangover severity. J. Clin. Med. 9, 786. 10.3390/jcm9030786 32183161 PMC7141364

[B24] Wachełko O. Zawadzki M. Szpot P. (2021). A novel procedure for stabilization of azide in biological samples and method for its determination (HS-GC-FID/FID). Sci. Rep. 11, 15568. 10.1038/s41598-021-95104-5 34330976 PMC8324859

[B25] World Health Organization (2024). Global status report on alcohol and health and treatment of substance use disorders. Available online at: https://www.who.int/publications/i/item/9789240096745 (Accessed June 25, 2024).

[B26] Yokoyama A. Yokoyama T. Mizukami T. Matsui T. Kimura M. Matsushita S. (2014). Blood ethanol levels of nonabstinent Japanese alcoholic men in the morning after drinking and their ADH1B and ALDH2 genotypes. Alcohol Alcohol 49, 31–37. 10.1093/alcalc/agt136 23969552

[B27] Yue R. Yang T. Niu D. Zeng Z. Wang X. Pan L. (2025). Integration of pharmacodynamics, network pharmacology and metabolomics to elucidate the effect and mechanism of Jingfang Granule in the treatment of Paraquat induced Pulmonary fibrosis. PLoS One 20, e0318246. 10.1371/journal.pone.0318246 39965011 PMC11835338

[B28] Zhu M. Zhou X. Zhao J. (2017). Quercetin prevents alcohol-induced liver injury through targeting of PI3K/Akt/nuclear factor-κB and STAT3 signaling pathway. Exp. Ther. Med. 14, 6169–6175. 10.3892/etm.2017.5329 29285175 PMC5740530

[B29] Zimatkin S. M. Anichtchik O. V. (1999). Alcohol-histamine interactions. Alcohol Alcohol 34 (2), 141–147. 10.1093/alcalc/34.2.141 10344773

